# Long-Term Dominance of Carbapenem-Non-Susceptible *Pseudomonas aeruginosa* ST111 in Hematologic Malignancy Patients and Hematopoietic Cell Transplant Recipients

**DOI:** 10.3389/fcimb.2022.904602

**Published:** 2022-06-16

**Authors:** Liyang Zhang, Filemon C. Tan, Lynne Strasfeld, Morgan Hakki, Natalia V. Kirienko

**Affiliations:** ^1^ Department of BioSciences, Rice University, Houston, TX, United States; ^2^ Division of Infectious Diseases, Department of Medicine, Oregon Health and Science University, Portland, OR, United States

**Keywords:** *P. aeruginosa*, hematopoietic cell transplant, hematologic malignancy, *C. elegans*, *oprD*, fitness

## Abstract

An epidemiological study uncovered that fluoroquinolone (FQ) neutropenic prophylaxis in hematopoietic cell transplant and hematologic malignancy (HCT/HM) patients was associated with breakthrough *Pseudomonas aeruginosa* bloodstream infections (BSIs) with isolates non-susceptible to both FQs and meropenem. The molecular epidemiology of the FQ/meropenem-non-susceptible *P. aeruginosa* isolates causing FQ-breakthrough BSIs in the HCT/HM patients remains unclear. Through whole genome sequencing on 57 P*. aeruginosa* isolates from 54 patients diagnosed with HM or receiving an HCT, we found that ST111 strains predominated, accounting for 22 (38.6%) of the isolates. 17 of 33 (51.5%) FQ-breakthrough BSIs were caused by ST111 strains, of which 15 (88.2%) were meropenem non-susceptible. ST111 strains, but not other *oprD*-deficient, meropenem-non-susceptible clinical strains, were found to have a colonization advantage over *P. aeruginosa* strain PA14 in *C. elegans* and to outcompete PA14 in *in vitro* co-culture assays. Together, we found that breakthrough *P. aeruginosa* BSIs during FQ prophylaxis in HCT/HM patients are dominated by clonally-related FQ/meropenem non-susceptible strains, predominantly ST111 type, and that the dominance of ST111 strains may be explained by a relative fitness advantage over other clinical strains. Additional work is necessary to better understand the factors driving the dominance and persistence of these ST111 strains.

## Introduction

Infections caused by Gram-negative bacteria, particularly drug-resistant pathogens, remain a significant cause of morbidity and mortality in neutropenic patients who are receiving chemotherapy for hematologic malignancies (HM) or hematopoietic cell transplant (HCT) recipients (Satlin and Walsh, 2017; Martinez-Nadal et al., 2020). In these patients, fluoroquinolone (FQ) prophylaxis is recommended (Taplitz et al., 2018), but the widespread use of FQ prophylaxis for these high-risk neutropenic patients has raised concerns about the emergence of resistant organisms (Rangaraj et al., 2010; Bow, 2011; Kern et al., 2018).

We previously described that FQ neutropenic prophylaxis in HCT and HM patients at Oregon Health and Science University (OHSU) was associated with breakthrough bloodstream infections (BSIs) caused by *P. aeruginosa* strains that were non-susceptible not only to FQs but also to meropenem, which was surprising since <5% of episodes were preceded by carbapenem exposure (Hakki et al., 2019). Whole genome sequencing (WGS) of 23 isolates obtained over an ~18 month period found that 6 of 13 meropenem-non-susceptible isolates sequenced belonged to multilocus sequence type (ST) 111, and 4 to ST446 (Hakki et al., 2019). These findings suggested that the selection of meropenem-non-susceptible *P. aeruginosa* isolates during FQ prophylaxis in HCT/HM patients at OHSU may arise, at least in part, from clonal dominance by closely-related, FQ- and meropenem-non-susceptible strains. However, the relatively small number of isolates available for WGS analysis, and the relatively short time period during which these isolates were collected, limited the conclusions that could be drawn about the molecular epidemiology of *P. aeruginosa* BSIs in these patients.

Here we expand on our previous findings to develop a better understanding of the role of dominant, clonal FQ/meropenem-non-susceptible *P. aeruginosa* isolates in causing breakthrough BSIs during FQ prophylaxis in HCT/HM patients and seek to gain a better understanding of the basis for dominance by meropenem-non-susceptible strains.

## Materials and Methods

### Bacterial Strains

*P. aeruginosa* PA14 is one of the two most frequently used lab strains of *Pseudomonas aeruginosa* for the study of virulence in this species (Mathee, 2018). *P. aeruginosa oprD*-deficient clinical isolates (M0025, M0067, M0101, M0103, M0117, M0128, 218M0087) were obtained from the OHSU Clinical Microbiology lab after being isolated from BSIs in OHSU HCT/HM patients. Transposon-insertion mutants PA14*oprD-1 and* PA14*oprD-2* came from commercially-available libraries (Jacobs et al., 2003; Liberati et al., 2006).

### *Caenorhabditis elegans* Strain Maintenance

A temperature-sensitive sterile strain of *C. elegans glp-4*(*bn2*) was used in this project (Beanan and Strome, 1992; Kirienko et al., 2013). Worms were maintained on nematode growth media (NGM) plates seeded with *E. coli* OP50. For propagation, *glp-4*(*bn2*) worms were incubated at 15°C. For experiments, synchronized L1 larvae *glp-4*(*bn2*) worms were grown overnight on concentrated OP50 lawn at room temperature, and then shifted to 25°C for 44~48 h to induce sterility prior to use.

### Clinical and Microbiological Data

*P. aeruginosa* isolates from BSIs in HCT/HM patients at OHSU occurring between 01 October 2016 and 30 June 2020 were obtained from the OHSU Clinical Microbiology Laboratory. Only the index isolate from each BSI episode was included in this study. The results of antimicrobial susceptibility testing, performed as part of routine clinical care by the OHSU microbiology laboratory using VITEK2 (bioMerieux, Durham, NC), were obtained by electronic medical record review. Results for cefepime, ceftazidime, meropenem, ciprofloxacin, piperacillin-tazobactam, gentamicin, and tobramycin were reported as susceptible, resistant, or intermediate, according to Clinical and Laboratory Standards Institute (CLSI) guidelines (CLSI, 2012; CLSI, 2019).

Institutional standards for FQ prophylaxis, and definitions of a BSI episode, FQ breakthrough BSI, and hospital-associated BSI have been previously published (Hakki et al., 2019; Fontana and Hakki, 2021).

### Whole Genome Sequencing

Bacterial DNA preparation, WGS, genome assembly, and MLST determination of each *P. aeruginosa* bloodstream isolate were performed as previously described (Hakki et al., 2019). Genomic sequences were deposited into GenBank (for accession numbers refer to **Supplementary Table 1**).

### Bacterial Growth Kinetics

An overnight bacterial culture was diluted in LB with or without antibiotics to a final OD600 0.1. 100 μl of diluted culture were added into each well of a 96-well plate, then covered with a BreatheEasy air-permeable membrane (Fisher Scientific) and was placed into a Cytation5 multimode plate reader (BioTek) for running growth kinetics at 37°C. For supernatant assay, an overnight bacterial culture was centrifuged and filtered through a 0.22 µm filter to yield bacteria-free spent media, referred to as “supernatant”. Growth kinetics were measured with 20% supernatant, v/v. At least three biological replicates were performed for each experiment.

### Slow-Killing Assay

Slow-Killing (SK) assays were performed as previously described (Kirienko et al., 2014). Briefly, 70 µl of *P. aeruginosa* overnight culture was spread evenly onto a 3.5 cm SK plate and grown at 37°C for 24 h, followed by another 24 h at 25°C. Fifty young adult *C. elegans* were transferred onto the *P. aeruginosa* lawn. Dead worms were removed and recorded daily until all worms in the control (PA14) were dead. Three biological replicates with ~150 worms/replicate were performed.

### Worm Colony-Forming Unit Assay

*P. aeruginosa* plates were prepared as for Slow-Killing assay. 15 adult worms were washed in a drop of S Basal and were picked into a 1.5 mL tube for a 10 min levamisole treatment (1% levamisole in S Basal (Acros Organics) w/v) after infection on SK plates at 25°C for 24 or 40 h, as specified. Worms were spun down at 1,000 rpm for 30 s and were washed 6 times with 1.2 ml wash buffer (S Basal + 0.01% Tween20, v/v). 10 µl of S Basal from the final wash were plated onto the LB plate (blank control). 200 µl worm lysate was obtained by vortexing for 1 min at maximum speed with 300 μl of zirconium beads (Fisher Scientific, 1.0 mm) added. Lysates were serially-diluted 5-fold and dilutions plated onto 10 cm LB plates containing appropriate antibiotics for selection (carbenicillin (300 ug/ml, Fisher Scientific) for dsRed plasmid-containing strains, gentamycin (15 ug/ml, Fisher Scientific) for PA14 transposon-insertion mutants, imipenem (1.5 ug/ml, Fisher Scientific) for carbapenem-resistant isolates, and rifampicin (50 ug/ml, Fisher Scientific) for PA14). The colonies were counted after a 24-hour incubation at 37°C and the colony-forming units (CFUs) per each worm were calculated. Each of three biological replicates included three technical replicates.

### *In Vitro* Competition Assay

PA14 and selected clinical isolates were mixed 1:1 based on the OD600. For the single-strain control groups, water was used instead of the second strain. After 24 h of incubation at 37°C, bacteria were washed off from plates using sterile water and were seeded onto antibiotic plates with serial 5-fold dilutions in a 96-well plate. The colonies were counted the next day. Three biological replicates were performed.

### Biofilm Quantification Assay

A single bacterial colony was inoculated into 5 mL LB medium and was incubated at 37°C overnight in a shaking incubator. The OD600 of each overnight culture (PA14, M0025, M0067, M0101, 218M0087, M0103, M0117, or M0128) was measured in a spectrophotometer. 0.5 mL M9 media (supplemented with 1 mM magnesium and 1 mM calcium) was added to each well of a 24-well plate. Based on OD600, the same amount of bacteria was added to each well. The plate was incubated at 30°C for 24 hours. The next day, media was aspirated from the plate with a pipette. 1 mL crystal violet solution (0.1% w/v crystal violet, 20% EtOH in water) was added to each well. The biofilm matrix was stained for 30 min at room temperature. Each well was washed with 0.5 mL PBS solution twice after staining. The plate was dried in the hood. To quantify the biofilm, crystal violet stain was resuspended in 0.5 mL of 30% acetic acid. The biofilm was measured at OD550 in a spectrophotometer (Kang et al., 2019).

## Results

### FQ/Meropenem-Non-Susceptible ST111 Isolates Predominate in FQ-Breakthrough *P. aeruginosa* BSIs in HCT/HM Patients

57 *P. aeruginosa* isolates, collected from 57 unique BSI episodes in 54 HCT/HM patients, were analyzed by WGS (**Supplementary Table 1**). Three patients had two unique BSI episodes separated by 120, 65, and 27 days. 48 of these 57 (84.2%) infections were classified as hospital-associated and 35 (61.4%) of the 57 isolates were classified as meropenem non-susceptible despite only 3 (5.3%) being isolated from patients with carbapenem exposure in the prior 180 days (**Supplementary Table 1**). ST111 strains were the most common single MLST, accounting for 22 (38.6%) of the 57 isolates (**Supplementary Table 1**). 21 of these 22 ST111 infections (95.5%) were classified as hospital-associated, and ST111 accounted for 43.7% (21/48) of all hospital-associated *P. aeruginosa* BSIs in this study. Over the approximately 4 years of this study, ST111 strains comprised a relatively stable portion of *P. aeruginosa* BSIs in HCT/HM patients, averaging at 38.6% (**Supplementary Figure 1**). The next largest contributor was the ST446 type, which included 13 infections (22.8% of all BSIs), all of which were hospital-associated (27.1% of the 48 total HAIs). Notably, 12 P*. aeruginosa* isolates causing hospital-associated BSIs in non-HCT/HM patients at OHSU were collected during the study period; none were due to ST111 or ST446 strains (**Supplementary Table 1**).

Of 33 episodes of FQ-breakthrough BSIs in HCT/HM patients, 17 (51.5%) were due to ST111 strains, 11 (33.3%) were ST446 strains, and the remaining 5 (15.1%) were caused by other MLST strains, with no other MLST accounting for more than 2 episodes (**Table 1**). Of the 17 ST111 FQ-breakthrough infections, 16 (94.1%) were FQ-non-susceptible and 15 (88.2%) were meropenem-non-susceptible. Similarly, of the 11 ST446 FQ-breakthrough BSIs, 10 (91%) were FQ-non-susceptible and 10 (91%) were meropenem-non-susceptible. All but two meropenem-non-susceptible ST111 strains (M0134 and M0177, both “intermediate” classification) and all ST446 strains had mutations in the *oprD* gene (**Supplementary Table 1**). Several mutations were consistent across ST111 strains, the most common being a premature stop codon at amino acid 100 (S100*) in 11 of the 17 (64.7%) strains. All meropenem-non-susceptible ST446 strains were characterized by a premature stop codon at amino acid 328. (Y328*). In comparison, most (17 of 24, 70.8%) of the isolates that did not break through FQ prophylaxis (while patients were receiving either a non-fluoroquinolone antibiotic or no antibiotic) were meropenem-susceptible, non-ST111 or ST446 strains, with no single MLST accounting for more than 1 isolate in this group.

**Table 1:** Molecular epidemiology of FQ breakthrough BSIs in HCT and HM patients.

	FQ breakthrough (N = 33)	Non FQ breakthrough (N = 24)
ST111, N (%)	17 (51.5)	5 (20.8)
FQ-NS	16	5
MEM-NS	15	5
ST446, N (%)	11 (33.3)	2 (8.3)
FQ-NS	10	2
MEM-NS	10	2
other, N (%)^a^	5 (15.1)	17 (70.8)
FQ-NS	2	2
MEM-NS	2	3

BSI, bloodstream infection; HCT, hematopoietic cell transplant; HM, hematologic malignancy; FQ, fluoroquinolone; MEM, meropenem; NS, non-susceptible. ST, sequence type.

a No single ST accounted for > 2 BSIs in either category.

These results are suggestive of clonal dominance by FQ-non-susceptible, meropenem-non-susceptible *P. aeruginosa* strains, particularly from the ST111 MLST, among HCT/HM patients receiving FQ prophylaxis. This clonal dominance likely contributes to our previous findings associating meropenem non-susceptibility with breakthrough infections during FQ prophylaxis (Hakki et al., 2019).

### Loss of OprD From PA14 Confers a Fitness Advantage in *C. elegans*

OprD loss has been shown to result in a fitness advantage over wild-type (WT) *P. aeruginosa* in a mouse model of co-infection, resulting in dramatic increases in mucosal colonization and systemic spread during neutropenia (Skurnik et al., 2013). We hypothesized that the dominance of meropenem-non-susceptible isolates in our HCT/HM patients in the absence of selective pressure for carbapenem resistance may be due to a relative fitness advantage conferred by inactivating *oprD* mutations present in these clinical isolates.

The nematode *C. elegans* is a commonly used model organism for studying the pathogenesis of *P. aeruginosa* because it has broad conservation of innate immunity and an array of infection models that recapitulate several important aspects of mammalian pathogenesis (Alper et al., 2007; Maadani et al., 2007; Pukkila-Worley et al., 2009; Engelmann and Pujol, 2010; Utari and Quax, 2013). It represents a simple, quick, and inexpensive host that can be used for medium- to high-throughput techniques to study the pathogenesis of various *oprD* mutants in the context of an intact host (Anderson et al., 2018).

**Figure 1:**
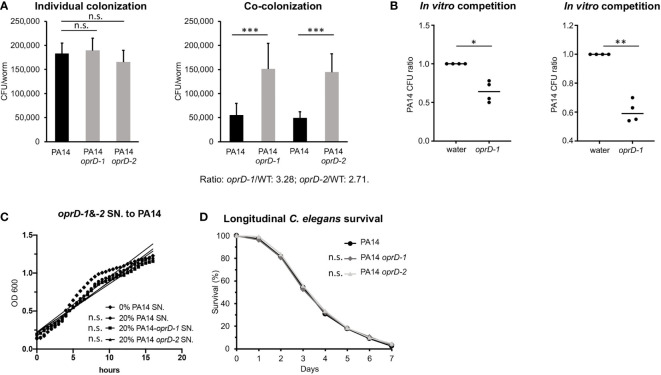
Impact of *oprD* deletion on colonization and virulence during *P. aeruginosa* infection. **(A)** The CFUs (colony-forming units) of WT *P. aeruginosa* PA14 or isogenic *oprD* transposon-insertion mutants PA14*oprD-1* or *-2* in worm intestinal lumen after an individual infection or co-infection for 40 hours. **(B)** Relative abundance of PA14::dsRED after 24 h growth in the presence of water or PA14*oprD-1* or *-2*. **(C)** Slopes of PA14 growth curve in the presence of 20% PA14 supernatant (control) or 20% PA14*oprD-1* or *-2* supernatants. **(D)** Longitudinal *C. elegans* survival during the infections with WT PA14 or PA14*oprD-1* or *-2*. Three biological replicates were performed. *p*-values were calculated using Student’s **t**-test **(A)** or one sample *t* and Wilcoxon tests **(B)**, or log-rank test **(D)**. n.s. *p* > 0.05; *p < 0.05; **p < 0.01; ***p < 0.001.

To evaluate the fitness of *oprD* mutants, synchronized, young adult *glp-4(bn2) C. elegans* worms were infected with either WT *P. aeruginosa* PA14 or one of two independently-derived PA14 mutants with transposon insertion into *oprD* (PA14*oprD*-1, PA14*oprD*-2). Each *oprD* transposon mutant showed *C. elegans* colonization comparable to WT PA14, suggesting that *oprD* disruption did not impair growth or infectivity (**Figure 1A**). Next, the assay was repeated with a 1:1 mixture of WT and PA14*oprD*-1 or PA14*oprD*-2. Differences in antimicrobial resistance were used to count colonies of each on selective plates. In this context, both *oprD* transposon mutants demonstrated significantly greater colonization (approximately three-fold higher) than WT PA14 (**Figure 1A**). Further, we demonstrated that WT PA14 grown in the presence of the *oprD* mutants grew less *in vitro*, suggesting that the *oprD* mutants limit the growth of the WT strain (62% of PA14 alone, on average, **Figure 1B**). Addition of supernatant from PA14*oprD-1* or *-2* did not impact WT PA14 growth (**Figure 1C**).

To further rule out a difference in colonization rate, we compared death in a *C. elegans* Slow-Killing (SK) assay between hosts infected with WT and *oprD* mutants (Tan et al., 1999). Although the precise mechanism of pathogenesis remains unclear in this assay, killing has been linked to colonization of the intestine (Utari and Quax, 2013; Kirienko et al., 2014). If the *oprD* mutants were growing significantly faster, they would be expected to demonstrate increased killing in the SK assay. Instead, no significant difference was seen in virulence between WT PA14 and *oprD* mutants (**Figure 1D**).

Overall, these data support the conclusion that the loss of *oprD* confers a growth advantage over the WT strain in co-infection studies. Importantly, by recapitulating what has been observed in murine co-infection models (Skurnik et al., 2013), these data validate *C. elegans* as a model for studying this aspect of the *P. aeruginosa* host-pathogen interaction (Kumar et al., 2020).

### ST111 *oprD*-Deficient Clinical Isolates Outcompete PA14 in *C. elegans* Models

Next, 7 FQ/meropenem-non-susceptible, *oprD*-mutant BSI isolates from OHSU HM/HCT patients (ST111/M0067, ST111/M0101, ST111/218M0087, ST111/M0025, ST291/M0103, ST299/M0128, and ST446/M0117, see **Table 2**) were evaluated to determine whether the colonization advantage seen in *oprD* transposon mutants was also observed in the clinical isolates.

**Table 2:** Co-colonization of *oprD*-deficient clinical isolates and PA14 in *C. elegans*.

Individual colonization					Co-colonization		
	OprD mutation	MLST	CFU/worm	*p* value		CFU/worm	*p* value
PA14			125,764	-	PA14	23,179	-
					M0067	87,839	<0.05
M0067	Ser100*	ST111	30,241	<0.001	PA14	19,537	-
M0101	Tyr121fs	ST111	48,299	<0.001	M0101	56,250	<0.05
218M0087	Tyr234fs	ST111	29,318	<0.001	PA14	28,519	-
M0025	Ser100*	ST111	45,578	<0.001	218M0087	55,093	<0.05
PA14			87,592	-	PA14	50,532	-
M0103	Gly162fs	ST291	29,719	<0.001	M0025	117,816	<0.001
M0128	Trp339*	ST299	99,198	n.s.	PA14	87,870	-
PA14			69,256	-	M0103	136	<0.05
M0117::RED	Tyr328*	ST446	57,654	n.s.	PA14	59,537	-
					M0117::RED	945	<0.001
					PA14	77,037	-
					M0128	254	<0.001

*Indicates stop codon.

fs, frameshift; MLST, multilocus sequence type; n.s., p > 0.05.

As expected, the *oprD*-mutant clinical isolates (except M0117, which was partially inhibited) grew well on 1.5 µg/ml imipenem (**Supplementary Figure 2**). Unlike the *oprD* mutants, PA14 growth was strongly inhibited by imipenem (**Supplementary Figure 2**). PA14 was able to grow on 50 ug/ml rifampicin, which the clinical isolates were susceptible to (**Supplementary Figure 2**) (Hu et al., 2016). This difference in susceptibility enabled differential plating on antimicrobials and allowed quantification of each isolate after recovery from co-infection assays.

Despite colonizing *C. elegans* more poorly than PA14 during individual infections, all four ST111 isolates outcompeted PA14 in co-infection assays (**Table 2**). In contrast, all three non-ST111 isolates were outcompeted by PA14 during co-infection despite two of these, M0128 and M0117, demonstrating equivalent colonization to PA14 during individual infections (**Table 2**). These results indicate that ST111 isolates are unique compared to other, non-ST111, meropenem non-susceptible *oprD*-mutant clinical isolates in exhibiting colonization dominance during co-infection with PA14.

### ST111 *oprD*-Deficient Clinical Isolates Increase *C. elegans* Survival During Co-Infection With PA14

**Figure 2:**
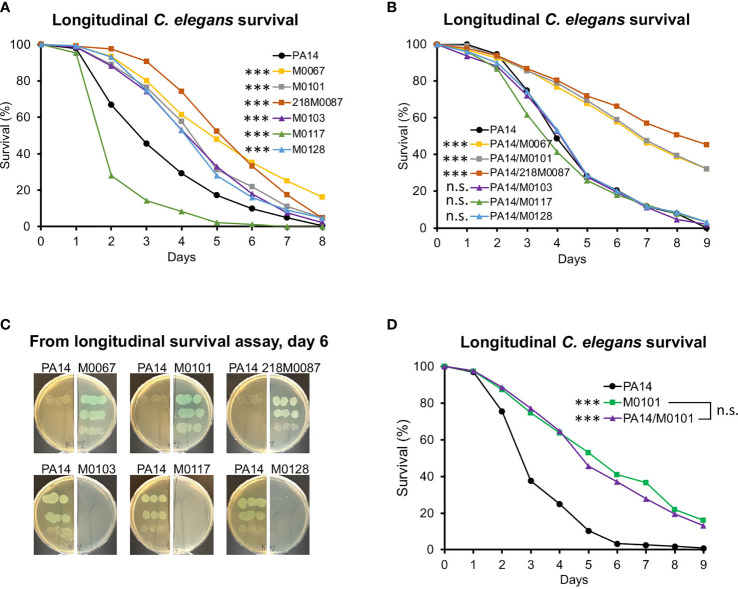
Virulence of *oprD*-deficient clinical strains in *C. elegans*. **(A)** Longitudinal *C. elegans* survival during the infection with either PA14 or *oprD*-deficient clinical isolates. **(B, D)** Longitudinal *C. elegans* survival during co-infection with PA14 or *oprD*-deficient clinical isolates. **(C)** The relative growth of PA14 and *oprD*-deficient clinical isolates on infection plates at day 6. Three biological replicates were performed. *p*-values were calculated using log-rank test. n.s. *p* > 0.05; ***p < 0.001.

To determine the consequences of colonization by the *oprD* mutant clinical isolates, young adult *glp-4(bn2) C. elegans* worms were infected with each isolate using the SK assay and survival was monitored. All clinical isolates, with the exception of M0117, were attenuated compared to PA14 (**Figure 2A**). Therefore, colonization fitness as measured in individual infection and pathogenesis appear to be unlinked in this assay.

Since co-infection with PA14 had restored colonization by the ST111 *oprD* mutant isolates, we tested whether co-infection would affect killing in the SK assay. Worms were infected with a 1:1 mixture of one of the ST111 isolates and PA14. Strikingly, virulence remained low, suggesting that reduction in virulence seen in these isolates was not solely due to reduced colonization (**Figure 2B**). In contrast, SK infections with mixtures of ST299/M0128, ST291/M0103, or ST446/M0117 with PA14 matched the outcome of infection with PA14 alone, negating the virulence characteristics seen for each clinical isolate alone (**Figure 2B**).

Plating bacteria after 6 d of co-infection, we observed the virulence phenotype in the SK co-infection assay was consistent with the dominant colonizing isolate (**Figure 2C**). The changes in virulence observed were not due to dilution of PA14, since no significant difference in survival was observed between M0101 alone and M0101 mixed with PA14 (ratio 1:1) (**Figure 2D**).

The data provide further evidence that the ST111 *oprD* mutants, like the transposon PA14*oprD* mutants, outcompete PA14 during co-infection of *C. elegans*. In contrast, all three non-ST111 isolates were outcompeted by PA14.

### ST111 Isolate Inhibition of PA14 Growth Requires the Presence of Bacterial Cells

**Figure 3:**
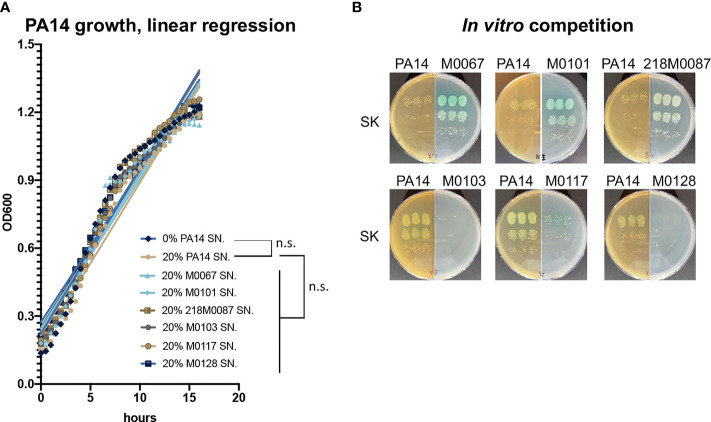
PA14 growth in supernatants of *oprD*-deficient clinical strains isolates and during *in vitro* co-culture with *oprD*-deficient clinical strains. **(A)** Slopes of PA14 growth curve in the presence of 20% PA14 supernatant (control) or 20% supernatant from *oprD*-deficient clinical isolates. **(B)** Growth of *oprD*-deficient clinical isolates or PA14 following an *in vitro* competition. Three biological replicates were performed. n.s. *p* > 0.05.

To investigate the mechanism of the fitness advantage of ST111 isolates, PA14 was incubated with supernatant from clinical isolates grown in culture (bacteria-free spent growth media). None of the supernatant samples affected PA14 growth (**Figure 3A**). In contrast, when media was inoculated with either PA14 alone or PA14 together with the *oprD*-mutant clinical isolates, only ST111 isolates (M0067, M0101, 218M0087) appeared to outcompete, or inhibit the growth of PA14, whereas the non-ST111 isolates (M0103, M0117, M0128) were outcompeted by PA14 (**Figure 3B**). These results indicate that living cells are required by ST111 isolates to inhibit PA14 growth during co-infection.

## Discussion

We previously reported that FQ prophylaxis in neutropenic HCT/HM patients at OHSU resulted in the selection of *P. aeruginosa* BSIs caused by meropenem-non-susceptible strains, despite the absence of selective pressure for carbapenem resistance (Skurnik et al., 2013). WGS of a small number of isolates revealed several clusters of closely-related meropenem-non-susceptible isolates from different patients, most frequently from the ST111 and ST446 MLSTs. This work expanded on those findings in order to gain better understanding of the potential contribution of clonal relatedness to the meropenem-non-susceptible phenotype of FQ-breakthrough *P. aeruginosa* BSIs in these patients.

The data from WGS presented here expand on our previous work and confirms the dominance of FQ/meropenem non-susceptible ST111 *P. aeruginosa*, and to a lesser extent ST446, causing breakthrough BSIs during FQ prophylaxis. Together, these two STs accounted for 84.8% of FQ-breakthrough BSIs, 89% of which were meropenem non-susceptible. Thus, our previous findings that FQ prophylaxis is associated with *P. aeruginosa* breakthrough BSIs with meropenem non-susceptible isolates is likely due in large part to the clonal dominance of these two FQ/meropenem non-susceptible STs in the HCT/HM patient population at OHSU.

Since the dominance of these meropenem non-susceptible strains is not due to ongoing selective pressure for carbapenem resistance, we analyzed whether inactivating mutations in *oprD*, which were present in almost all meropenem non-susceptible isolates (**Supplementary Table 1**), may confer a relative fitness advantage, as has been shown in mice (Skurnik et al., 2013). Using a *C. elegans* model of infection that involved intestinal colonization, we found that loss of OprD function in the reference PA14 strain resulted in a fitness advantage compared to the WT isogenic control in co-infection experiments but not individual infection. The basis for these findings remains unclear. OprD mediates the cell entry of carbapenems, amino acids, and peptides (Li et al., 2012; Chevalier et al., 2017). *oprD*-deficient mutants have been shown to be less susceptible to acidic pH and to the bactericidal effects of human serum (Skurnik et al., 2013). *C. elegans*’ intestinal lumen is also acidic, ranging from ~6 at the anterior to ~3.5 at the posterior (Chauhan et al., 2013). However, while this resistance may be relevant, it does not explain why the *oprD* mutants did not exhibit a colonization advantage during individual infection in the absence of wild-type PA14. Additional work is required to determine the mechanistic basis by which loss of functional OprD results in a fitness advantage. Regardless, these results may inform decisions pertaining to the role of carbapenems as empiric therapy for febrile neutropenia and their use in other clinical contexts, since, as now shown by two independent groups using two different model systems (Freifeld et al., 2011; Skurnik et al., 2013), the selective pressure exerted by carbapenems on *oprD* appears to result in *P. aeruginosa* with enhanced fitness characteristics.

However, of the FQ/meropenem non-susceptible *oprD*-deficient clinical isolates tested, only ST111 isolates exhibited a fitness advantage over WT PA14 during co-infection assays. This finding likely explains the numeric dominance of ST111 isolates during FQ breakthrough BSIs in our HCT/HM patients. Co-culture with ST111 strains markedly reduced PA14 growth, whereas supernatant from ST111 cultures had no impact on PA14 growth, suggesting that the fitness advantage of ST111 isolates is due to mechanisms dependent on the presence of live bacterial cells.

ST111 is one of three major international “high-risk” *P. aeruginosa* clones, along with ST175 and ST235. These groups are known for causing outbreaks of multidrug or extremely drug-resistant infections in healthcare settings, including HCT units (Cabot et al., 2012; Oliver et al., 2015; Kossow et al., 2017). In contrast to our findings, previous work did not demonstrate a competitive advantage of ST111 strains when co-cultured *in vitro* with PAO1, another *P. aeruginosa* reference strain (Mulet et al., 2013). The ST111 isolates in this study may be unique with regards to that property. Alternatively, this difference could relate to variations in virulence that have been exhibited by different strains of *P. aeruginosa* (Lee, et al., 2006). Detailed genotypic and phenotypic analyses of the ST111 isolates in this study will be required to define the mechanism conferring their fitness advantage. The increased capacity of these ST111 isolates to form biofilm (**Supplementary Figure 3**) may contribute to their persistence and clonal dominance, as may other qualities to be analyzed such as motility and spontaneous mutation rates may also play roles (Mulet et al., 2013). An initial genomic analysis, while far from complete, revealed that almost all ST111 clinical isolates contained a premature stop codon at amino acid position 152 in the major quorum sensing regulatory gene *lasR* (Venturi, 2006), whereas all other clinical isolates contained wild-type *lasR* genes (**Supplementary Figure 4**). *lasR* mutations have been shown to confer increased fitness under certain conditions (Heurlier et al., 2006; Diggle et al., 2007; Sandoz et al., 2007; Clay et al., 2020). Future work will evaluate the potential role of *lasR* in our findings.

Loss of functional OprD in non-ST111 clinical isolates, including ST446 isolates, was not associated with a relative fitness benefit, highlighting what is likely the complex contribution of numerous factors, including other drug resistance mutations (Geisinger and Isberg, 2017), toward overall fitness under various conditions (Basta et al., 2017; Schinner et al., 2020). The explanation for why FQ/meropenem non-susceptible ST446 isolates comprise a significant proportion of FQ breakthrough BSIs, despite a relative fitness disadvantage compared to PA14, is unclear. PA14 may not be representative of other FQ-non-susceptible clinical isolates that would be selected for during FQ prophylaxis. Experiments comparing relative fitness between clinical isolates are in process.

Since 95% (21 of 22) of ST111 BSIs were classified as hospital associated, consistent with most FQ prophylaxis in these high-risk patients being administered in the hospital, it is likely that ST111 isolates also dominate in an environmental reservoir on the OHSU HCT/HM unit. This presumably contributes to patient colonization during hospitalization as a first step towards invasive disease such as a BSI (Gomez-Zorrilla et al., 2015; Nesher et al., 2015; Harris et al., 2016; Cohen et al., 2017). Efforts are underway to define the *P. aeruginosa* populations in various environmental niches on the HCT/HM unit and to determine the genetic relatedness of those environmental populations with isolates from BSIs.

In summary, we found that breakthrough *P. aeruginosa* BSIs during FQ prophylaxis in HCT/HM patients are dominated by clonally-related FQ/meropenem non-susceptible isolates, predominantly from the ST111. The dominance of ST111 isolates may be explained by their relative fitness advantage over other FQ/meropenem non-susceptible clinical isolates. The mechanism driving this advantage is unclear, but appears to require living bacteria. Additional work is necessary to better understand the factors driving the dominance and persistence of these ST111 isolates.

## Data Availability Statement

The datasets presented in this study can be found in online repositories. The names of the repository/repositories and accession number(s) can be found in the article/**Supplementary Material**.

## Author Contributions

NK and MH contributed to conception and design of the study. LZ, FT, LS conducted experiments, organized the database, and performed the statistical analysis. LZ, NK, MH drafted the manuscript. NK and MH funded the research and provided the overall supervision of the project. All authors contributed to manuscript revision, read, and approved the submitted version.

## Funding

The study was supported by the CFF Pilot and Feasibility grant KIRIEN20I0 to NVK and CFF Traineeship award 002287H221 to LZ, and OHSU Core Pilot Fund Awards to MH and LS. The funders had no role in study design, data collection and analysis, decision to publish, or preparation of the manuscript.

## Conflict of Interest

The authors declare that the research was conducted in the absence of any commercial or financial relationships that could be construed as a potential conflict of interest.

## Publisher’s Note

All claims expressed in this article are solely those of the authors and do not necessarily represent those of their affiliated organizations, or those of the publisher, the editors and the reviewers. Any product that may be evaluated in this article, or claim that may be made by its manufacturer, is not guaranteed or endorsed by the publisher.
