# Supplementary material for: Long-Term Dominance of Carbapenem-Non-Susceptible Pseudomonas aeruginosa ST111 in Hematologic Malignancy Patients and Hematopoietic Cell Transplant Recipients
Source: Front Cell Infect Microbiol. 2022 Jun 16;12:904602. doi: 10.3389/fcimb.2022.904602 (PMC9244782; doi:10.3389/fcimb.2022.904602)
Supplement: Supplementary file 1 [file DataSheet_1.pdf]

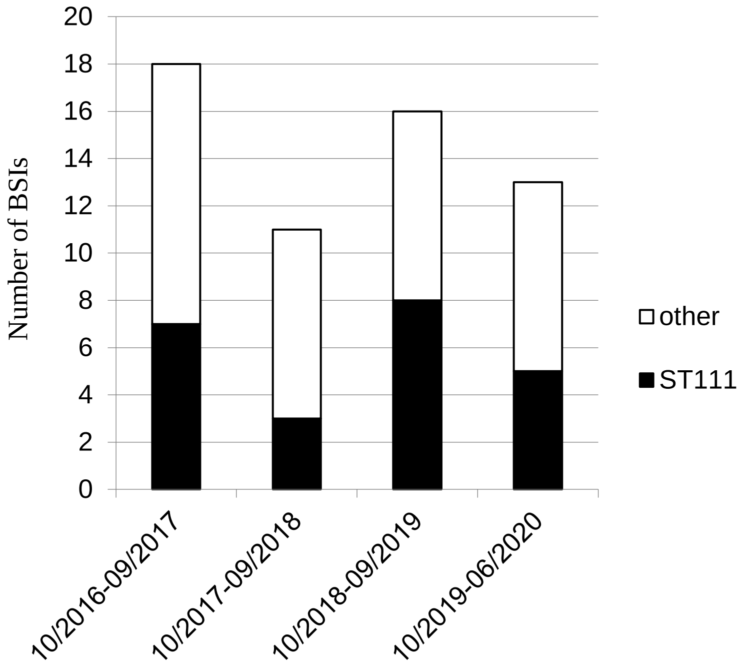

Supplemental Figure 1. BSIs caused by ST-111 strains in HCT/HM patients each year during the study period.

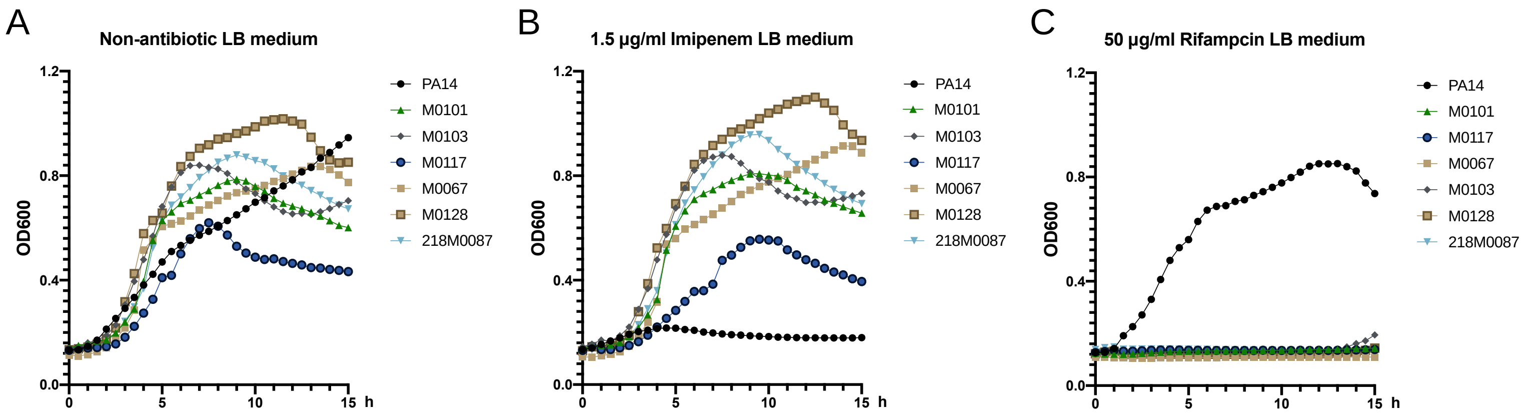

Supplemental Figure 2. The growth curve of PA14 and *oprD* isolates with or without antibiotics. PA14 and *oprD* isolates were incubated in non-antibiotic LB medium (**A**), 1.5 μg/ml imipenem LB medium (**B**), and 50 μg/ml rifampicin LB medium for 16 hours (**C**). There biological replicates were performed.

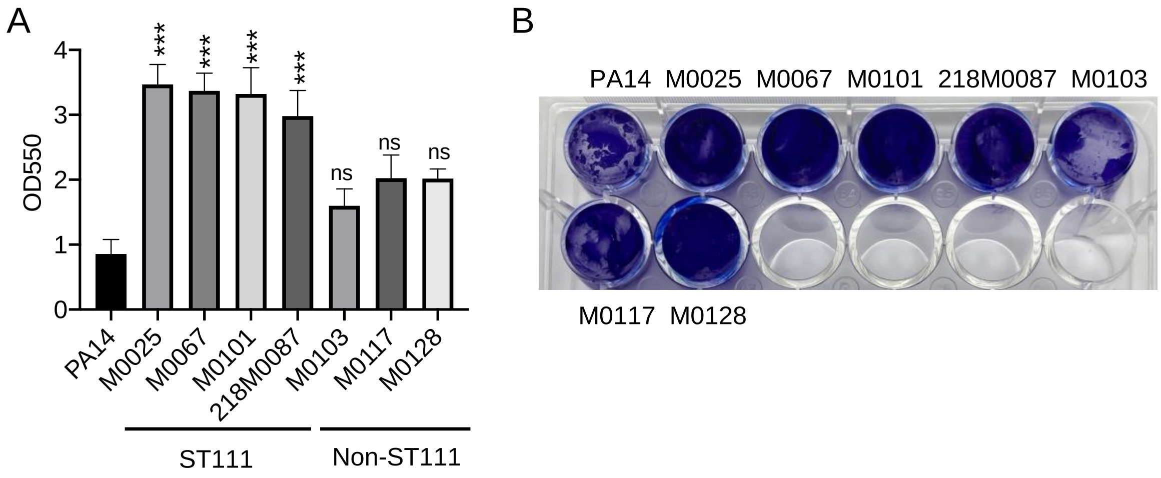

Supplemental Figure 3. The biofilm formation of PA14, ST111, and non-ST111 strains. PA14 and *oprD* isolates were incubated in M9 media (supplemented with magnesium and calcium) for 24 hours. Quantification (**A**) and representative images (**B**) are shown. Three biological replicates were performed. *p*-values were calculated using ANOVA with Dunnett's multiple comparisons test. n.s. $p > 0.05$; *** $p < 0.001$.

```
PAO1         MALVDGFLELERSSGKLEWSAILQKMASDLGFSKILFGLLPKDSQDYENAFIVGNYPAAW  60
PA14         MALVDGFLELERSSGKLEWSAILQKMASDLGFSKILFGLLPKDSQDYENAFIVGNYPAAW  60
M0025        MALVDGFLELERSSGKLEWSAILQKMASDLGFSKILFGLLPKDSQDYENAFIVGNYPAAW  60
218M0087     MALVDGFLELERSSGKLEWSAILQKMASDLGFSKILFGLLPKDSQDYENAFIVGNYPAAW  60
M0101        MALVDGFLELERSSGKLEWSAILQKMASDLGFSKILFGLLPKDSQDYENAFIVGNYPAAW  60
M0067        MALVDGFLELERSSGKLEWSAILQKMASDLGFSKILFGLLPKDSQDYENAFIVGNYPAAW  60
M0117        MALVDGFLELERSSGKLEWSAILQKMASDLGFSKILFGLLPKDSQDYENAFIVGNYPAAW  60
M0103        MALVDGFLELERSSGKLEWSAILQKMASDLGFSKILFGLLPKDSQDYENAFIVGNYPAAW  60
M0128        MALVDGFLELERSSGKLEWSAILQKMASDLGFSKILFGLLPKDSQDYENAFIVGNYPAAW  60
             ************************************************************

PAO1         REHYDRAGYARVDPTVSHCTQSVLPIFWEPSIYQTRKQHEFFEEASAAGLVYGLTMPLHG  120
PA14         REHYDRAGYARVDPTVSHCTQSVLPIFWEPSIYQTRKQHEFFEEASAAGLVYGLTMPLHG  120
M0025        REHYDRAGYARVDPTVSHCTQSVLPIFWEPSIYQTRKQHEFFEEASAAGLVYGLTMPLHG  120
218M0087     REHYDRAGYARVDPTVSHCTQSVLPIFWEPSIYQTRKQHEFFEEASAAGLVYGLTMPLHG  120
M0101        REHYDRAGYARVDPTVSHCTQSVLPIFWEPSIYQTRKQHEFFEEASAAGLVYGLTMPLHG  120
M0067        REHYDRAGYARVDPTVSHCTQSVLPIFWEPSIYQTRKQHEFFEEASAAGLVYGLTMPLHG  120
M0117        REHYDRAGYARVDPTVSHCTQSVLPIFWEPSIYQTRKQHEFFEEASAAGLVYGLTMPLHG  120
M0103        REHYDRAGYARVDPTVSHCTQSVLPIFWEPSIYQTRKQHEFFEEASAAGLVYGLTMPLHG  120
M0128        REHYDRAGYARVDPTVSHCTQSVLPIFWEPSIYQTRKQHEFFEEASAAGLVYGLTMPLHG  120
             ************************************************************

PAO1         ARGELGALSLSVEAENRAEANRFMESVLPTLWMLKDYALQSGAGLAFEHPVSKPVVLTSR  180
PA14         ARGELGALSLSVEAENRAEANRFMESVLPTLWMLKDYALQSGAGLAFEHPVSKPVVLTSR  180
M0025        ARGELGALSLSVEAENRAEANRFMESVLPTL-----------------------------  151
218M0087     ARGELGALSLSVEAENRAEANRFMESVLPTL-----------------------------  151
M0101        ARGELGALSLSVEAENRAEANRFMESVLPTL-----------------------------  151
M0067        ARGELGALSLSVEAENRAEANRFMESVLPTL-----------------------------  151
M0117        ARGELGALSLSVEAENRAEANRFMESVLPTLWMLKDYALQSGAGLAFEHPVSKPVVLTSR  180
M0103        ARGELGALSLSVEAENRAEANRFMESVLPTLWMLKDYALQSGAGLAFEHPVSKPVVLTSR  180
M0128        ARGELGALSLSVEAENRAEANRFMESVLPTLWMLKDYALQSGAGLAFEHPVSKPVVLTSR  180
             *******************************

PAO1         EKEVLQWCAIGKTSWEISVICNCSEANVNFHMGNIRRKFGVTSRRVAAIMAVNLGLITL   239
PA14         EKEVLQWCAIGKTSWEISVICNCSEANVNFHMGNIRRKFGVTSRRVAAIMAVNLGLITL   239
M0025        -----------------------------------------------------------   151
218M0087     -----------------------------------------------------------   151
M0101        -----------------------------------------------------------   151
M0067        -----------------------------------------------------------   151
M0117        EKEVLQWCAIGKTSWEISVICNCSEANVNFHMGNIRRKFGVTSRRVAAIMAVNLGLITL   239
M0103        EKEVLQWCAIGKTSWEISVICNCSEANVNFHMGNIRRKFGVTSRRVAAIMAVNLGLITL   239
M0128        EKEVLQWCAIGKTSWEISVICNCSEANVNFHMGNIRRKFGVTSRRVAAIMAVNLGLITL   239
```

Supplemental Figure 4. The alignment of LasR protein. The sequences of LasR protein from PA14, PAO1, ST111 strains (M0025,M0067, M0101, 218M0087), and non-ST111 strains (M0103, M0117, M0128)) is shown.
